# Socioeconomic differences in discharge against medical advice and hospital admission among emergency department visits associated with substance use in the United States

**DOI:** 10.3389/fpubh.2025.1431384

**Published:** 2025-05-08

**Authors:** Zahra Mojtahedi, Pearl Kim, Ji Yoo, Binglong Wang, Jay J. Shen

**Affiliations:** ^1^Department of Healthcare Administration and Policy, School of Public Health, University of Nevada, Las Vegas, NV, United States; ^2^Research Core Capacity, Northern Arizona University, Flagstaff, AZ, United States; ^3^School of Medicine, University of Nevada, Las Vegas, NV, United States; ^4^Department of Healthcare Administration, Asia University, Taichung, Taiwan; ^5^Center for Health Disparities Research, School of Public Health, University of Nevada, Las Vegas, NV, United States

**Keywords:** discharge against medical advice, disparity, emergency department, inpatient admission, public health

## Abstract

**Background:**

Discharge against medical advice (DAMA) and inpatient admission (IA) among emergency department (ED) visits are two important outcomes in hospital utilization, while the first one has been mainly considered a negative outcome.

**Aims:**

This study aimed to examine the association of socioeconomic factors with DAMA and IA among ED visits with substance use (age 12–64 years) before and after the COVID-19 pandemic.

**Methods:**

The study retrospectively analyzed the Nationwide Emergency Department Sample (NEDS) from 2019 to 2020. The International Classification of Diseases 10th Revision (ICD-10) codes were used to identify opioid, cannabis, and alcohol use, and smoking.

**Results:**

The pandemic was significantly associated with higher odds of IA (OR 1.04, CI 1.02–1.06). Female gender and rural hospitals were adversely associated with both DAMA and IA, but lower household incomes were positively and negatively associated with DAMA and IA, respectively. Race and health insurance were partly differently associated with these outcomes. Asian patients exhibited significantly lower odds (OR 0.82, CI 0.71–0.88) regarding DAMA. Black (OR 0.79, CI 0.78–0.80) and Native American patients (OR 0.87, CI 0.82–0.90) exhibited lower odds, and Hispanic (OR 1.05, CI 1.03–1.06) and Asian patients (OR 1.40, CI 1.33–1.44) had higher odds compared to White patients in terms of AI. Except for self-pay, which was associated with lower odds of IA, Medicaid, self-pay, and free care were significantly associated with higher odds of DAMA and IA. Our results also showed that the COVID-19 pandemic affected the association of health insurance with IA, but not with DAMA.

**Conclusion:**

These findings highlight the complex association of socioeconomic factors with DAMA and IA. By addressing these differences within the hospital setting, providers can mitigate the negative consequences of substance use on patient health and reduce the burden on healthcare systems.

## Introduction

Discharge against medical advice (DAMA), which occurs in approximately 2% of emergency department (ED) visits, and inpatient admission (IA), which occurs in approximately 13.1% of ED visits, are ED visit outcomes that play a crucial role in shaping evidence-based policies (1–3). ED-DAMA occurs when a patient decides to leave the ED before completing the recommended medical evaluation or treatment, against the advice of healthcare providers. ED-IA refers to cases where a patient seen in the emergency department is admitted to the hospital for further treatment and care (1–3). Both DAMA and IA during ED visits have been linked to socioeconomic status and substance use, challenges that might have been intensified by the impacts of the COVID-19 pandemic (4). ED visits involving substance use are estimated to be 1,838 per 100,000 population (5), highlighting the significant burden of substance use on emergency care. Addressing challenges associated with DAMA and IA, particularly those associated with substance use, is critical to mitigating substance use’s impact on patient health and reducing healthcare system burdens.

Discharges against medical advice and inpatient admission pose challenges for healthcare providers and hospitals, as well as patients (2–4, 6–9). Notably, DAMA can lead to hospital revenue loss and compromised care quality. Both DAMA and IA have been associated with socioeconomic status and hospital characteristics in addition to substance use (6–8). Patients’ decisions to be discharged were also probably impacted by the COVID-19 pandemic since they faced unprecedented challenges, notably fear of the virus. Moreover, the COVID-19 pandemic significantly reduced both ED visits and IA visits, but the proportion of visits for substance-use conditions increased (4, 10, 11). Emerging evidence also suggests a complex interaction of the COVID-19 pandemic with socioeconomic characteristics, including race/ethnicity and health insurance, leading to disproportionately adverse impacts on specific racial or ethnic groups (4, 8). Research is needed to provide updated information on socioeconomic factors associated with DAMA and IA during ED visits, particularly after the pandemic and its interactions with sociodemographic factors, in order to develop targeted interventions and policies aimed at improving healthcare outcomes for all individuals, particularly during a crisis.

The COVID-19 pandemic has profoundly affected healthcare delivery, notably altering patterns of ED utilization and outcomes related to substance use (4, 8). Studies have reported a significant increase in substance use-related ED visits during the pandemic period, highlighting the growing burden of substance use disorders on emergency care (11). Additionally, the pandemic’s impact on mental health and access to addiction treatment services has been profound, leading to increased challenges in managing substance use disorders within ED settings (4, 8, 11). Disruptions in healthcare access, financial instability, and social isolation further exacerbated these issues, disproportionately affecting marginalized populations. Racial and ethnic minorities, low-income individuals, and those without stable health insurance coverage faced greater barriers to receiving timely and adequate emergency care (8, 11). These disparities likely influenced patterns of ED discharge against DAMA and IA during the pandemic. Examining how COVID-19 shaped these relationships is essential for understanding healthcare inequities and informing policies aimed at mitigating disparities in ED outcomes during public health crises.

Despite the well-recognized importance of socioeconomic factors in ED visits involving substance use, there is a notable gap in the updated literature examining their role in the context of ED-DAMA and ED-IA, particularly during the COVID-19 pandemic. Investigating these associations provides valuable insights into actual disparities and informs targeted interventions aimed at improving healthcare equity and patient outcomes. Consequently, this study aimed to investigate the association of socioeconomic factors, including gender, age, race/ethnicity, health insurance, income, and hospital regions and types, with ED-DAMA and ED-IA associated with substance use in US hospitals. The associations of the COVID-19 pandemic with ED-DAMA and ED-IA were also investigated.

## Methods

### Data

This pooled cross-sectional study retrospectively analyzed the Nationwide Emergency Department Sample (NEDS), the largest all-payer emergency department database in the United States. NEDS provides data on over 30 million ED visits annually, representing a stratified sample of hospital-based EDs across the country. The database includes information on patient demographics, visit characteristics, and outcomes, making it a robust resource for examining trends and disparities in ED utilization (12). Further details on the design and methodology of NEDS are available from its publisher, the Healthcare Cost and Utilization Project (HCUP) (12). The study period spanned from January 1, 2019, to December 31, 2020, encompassing both pre-pandemic and pandemic periods. The age range of 12–64 years was selected because substance use is uncommon in children younger than 12, while patients over the age of 64 are more likely to use opioids for pain management rather than for recreational purposes or mental health issues. By excluding these groups, the analysis focuses on age groups most likely to present with substance-use-related concerns, thereby improving the specificity of the findings. A total of 3,778,386 ED visits were identified for analysis within the defined study period and age range. A total of 3,778,386 ED visits were included in the analysis within the defined study period and age range. Substance use was identified using International Classification of Diseases, 10th Revision (ICD-10) codes, as previously described (4) and detailed in Supplementary File 1. Substance use was not necessarily the primary reason for the ED visit. Among these visits, 1.0% were associated with opioid use, 1.9% with cannabis use, 3.9% with alcohol use, and 18.4% with nicotine use. In total, 820,053 ED visits were identified as being associated with the use of at least one of these four substances—opioids, cannabis, alcohol, or nicotine smoking. This subset of ED visits served as the sample for analysis.

### Measures

The analysis included two binary outcomes: ED-DAMA and ED-IA. Both outcomes were identified using variables available in the NEDS dataset, with ED-DAMA defined as patient-initiated discharge against medical advice and ED-IA as hospital admission following an ED visit. Predictors were year (2020 during the pandemic vs. 2019 pre-pandemic), sex (male, female), and age groups (12–17, 18–24, 25–34, 35–44, 45–54, and 55–64), race/ethnicity (White, Black, Hispanic, Asian, Native American, and others), health insurance (private insurance, Medicaid, self-pay, and free care), median household income based on patient ZIP codes (0–25th, 26th–50th, 51st–75th, and 76th–100th percentile), and hospital characteristics (hospital types: urban vs. rural and hospital geographic regions: Northeast, Midwest, South, and West). Interactions were evaluated for the year with race/ethnicity and health insurance to examine disparities in outcomes by the pandemic period.

### Statistical analyses

Statistical analyses were conducted using SAS version 9.4. Descriptive analyses were performed to summarize the distributions of key variables, including demographic characteristics, socioeconomic factors, and hospital-related features. Frequencies and percentages were reported for all variables. The results were stratified by outcomes (ED-DAMA and ED-IA) to provide an overview of differences across groups. Binary logistic regression analyses were conducted to examine factors associated with ED-DAMA and ED-IA. Predictors included year (during-pandemic, 2020 vs. pre-pandemic, 2019), sex, age, race/ethnicity, health insurance, median household income, and hospital types. Interaction terms with the pandemic (year 2020) were incorporated to evaluate whether the associations of ED-DAMA and ED-IA with race/ethnicity or health insurance differed during the COVID-19 pandemic. All models were adjusted for potential confounders, including hospital regions (Northeast, Midwest, South, and West). Missing data were automatically removed by the software during regression analyses. Statistical significance was determined using *p*-values of less than 0.05.

## Results

Table 1 displays a descriptive analysis of ED-DAMA and ED-IA associated with substance use (2019 and 2020 NEDS). For ED-DAMA, the age distribution showed that 0.3% of cases were among individuals aged 12–17, 9.4% were 18–24, 27.5% were 25–34, 26.1% were 35–44, 20.7% were 45–54, and 15.9% were 55–64. The racial/ethnic distribution was as follows: 61.3% of cases involved White patients, 23.0% were Black, 11.1% were Hispanic, 0.9% were Asian, and 0.7% were Native American. Regarding median household income for the patient’s ZIP code, 44.5% of cases were from ZIP codes in the lowest income bracket (0–25th percentile), 26.7% were in the 26th–50th percentile, 18.2% in the 51st–75th percentile, and 10.6% in the highest income bracket (76th–100th percentile). For ED-IA, older patients had higher IA rates, with 29.0% of cases occurring among those aged 55–64. The racial/ethnic breakdown showed that 23.6% of cases involved White patients, 10.6% were Black, 1.2% were Hispanic, 0.9% were Asian, and 2.8% were Native American. The distribution of median household income for the patient’s ZIP code was similar to ED-DAMA, with most cases (38.9%) coming from the lowest income bracket (0–25th percentile), followed by 26.4% in the 26th–50th percentile, 20.2% in the 51st–75th percentile, and 14.5% in the highest income group (76th–100th percentile).

**Table 1:** Descriptive analysis of discharge against medical advice and inpatient admission during emergency department visits with substance use (NEDS)*.

Factor	Discharge against medical advice (*N* = 20,434)	Inpatient admission (*N* = 71,503)
Year		
2019	10,477	71,272
2020	9,957	70,231
Gender (%)		
Male	53.4	60.9
Female	41.6	39.1
Age (%)		
12–17	0.3	0.8
18–24	9.4	6.8
25–34	27.5	17.9
35–44	26.1	21.0
45–54	20.7	24.5
55–64	15.9	29.0
Race/ethnicity (%)		
White	61.3	62.6
Black	23.0	20.4
Hispanic	11.1	11.7
Asian and Pacific Islander	0.9	1.5
Native American	0.7	0.9
Others	3.0	2.9
Health insurance (%)		
Private insurance	18.1	28.9
Medicaid	49.6	47.4
Self-pay (uninsured)	28.0	17.3
Free care	1.3	1.6
Others	1.1	4.8
Median household income for patient’s ZIP Code (%)		
0–25th percentile	44.5	38.9
26th to 50th percentile (median)	26.7	26.4
51st to 75th percentile	18.2	20.2
76th to 100th percentile	10.6	14.5
Hospital type (%)		
Urban	85.3	86.5
Rural	14.7	13.5
Hospital region (%)		
West	16.1	17.4
Northeast	23.2	22.8
Midwest	42.6	41.7
South	18.1	18.1

*NEDS, the Nationwide Emergency Department Sample.

Table 2 presents factors associated with ED-DAMA and ED-IA with substance use in US hospitals, based on the NEDS. The associations of the pandemic period (year, 2020), gender, age, race/ethnicity and its interaction with the pandemic, health insurance and its interaction with the pandemic, median household income for the patient’s ZIP code, hospital type, and hospital region with ED-DAMA as well as ED-IA were investigated.

**Table 2:** Factor associated with discharge against medical advice and inpatient admission among emergency department visits in US hospitals (NEDS*).

Factors	Discharge against medical advice		Admitted to inpatient care	
	OR (95% CI)	*p*-value	OR (95% CI)	*p*-value
Year				
2019 (ref)				
2020	1.04 (0.99–1.07)	0.1154	1.04 (1.02–1.06)	<0.0001
Gender				
Male (ref)				
Female	0.87 (0.84–0.89)	<0.0001	0.91 (0.90–0.92)	<0.0001
Age				
35–44 (ref)				
12–17	0.24 (0.18–0.28)	<0.0001	0.40 (0.37–0.42)	<0.0001
18–24	0.78 (0.73–0.80)	<0.0001	0.54 (0.52–0.55)	<0.0001
25–34	0.93 (0.90–0.95)	0.0010	0.71 (0.70–0.72)	<0.0001
45–54	0.98 (0.94–1.01)	0.4207	1.55 (1.52–1.57)	<0.0001
55–64	0.92 (0.88–0.94)	0.0004	2.40 (2.36–2.43)	<0.0001
Race/ethnicity				
White (ref)				
Black	1.01 (0.96–1.04)	0.5932	0.79 (0.78–0.80)	<0.0001
Hispanic	1.03 (0.97–1.06)	0.3500	1.05 (1.03–1.06)	<0.0001
Asian	0.82 (0.71–0.88)	0.0047	1.40 (1.33–1.44)	<0.0001
Native American	0.88 (0.76–0.96)	0.1036	0.87 (0.82–0.90)	<0.0001
yr2020*Black	1.01 (0.96–1.03)	0.7999	1.00 (0.98–1.01)	0.7275
yr2020*Hispanic	0.93 (0.88–0.96)	0.0121	0.98 (0.96–0.99)	0.1377
yr2020*Asian	0.93 (0.81–1.00)	0.2786	1.04 (1.00–1.07)	0.0755
yr2020*Native American	1.12 (0.97–1.21)	0.1311	1.05 (0.99–1.08)	0.0990
Health insurance				
Private insurance (ref)				
Medicaid	1.27 (1.22–1.30)	<0.0001	1.06 (1.04–1.07)	<0.0001
Self-pay (uninsured)	1.22 (1.17–1.25)	<0.0001	0.65 (0.64–0.66)	<0.0001
Free care	1.15 (1.04–1.22)	0.0087	1.43 (1.37–1.46)	<0.0001
yr2020* Medicaid	0.98 (0.94–1.00)	0.1881	1.02 (1.00–1.03)	0.0268
yr2020*self-pay	0.99 (0.96–1.02)	0.7807	1.07 (1.05–1.08)	<0.0001
yr2020* Free care	1.06 (0.96–1.13)	0.2608	0.92 (0.88–0.94)	0.0002
Median household income for patient’s ZIP Code				
76th to 100th percentile (ref)				
0–25th percentile	1.14 (1.11–1.16)	<0.0001	0.99 (0.98–1.00)	0.3302
26th to 50th percentile (median)	0.99 (0.96–1.00)	0.4828	0.97 (0.96–0.98)	<0.0001
51st to 75th percentile	0.98 (0.95–1.00)	0.2640	1.00 (0.99–1.01)	0.7789
Hospital type				
Urban (ref)				
Rural	0.81 (0.78–0.83)	<0.0001	0.79 (0.77–0.79)	<0.0001
Hospital region				
West (Ref)				
Northeast	1.07 (1.04–1.09)	<0.0001	0.97 (0.96–0.98)	0.0001
Midwest	0.90 (0.88–0.92)	<0.0001	0.96 (0.95–0.97)	<0.0001
South	1.03 (1.01–1.05)	0.0084	1.24 (1.23–1.25)	<0.0001

*NEDS, Nationwide Emergency Department Sample.

### Discharge against medical advice

Pandemic period (year, 2020) was associated with a non-significant increase in the odds of ED-DAMA (OR 1.04, 95% CI 0.99–1.07). Asian patients (vs. White patients) exhibited significantly lower odds of ED-DAMA (OR 0.82, 95% CI 0.71–0.88). The interaction between the pandemic period (year 2020) and race revealed that Hispanic patients (vs. White patients) during the pandemic had a significant decrease in the odds of ED-DAMA (OR 0.93, 95% CI 0.88–0.96). Medicaid, self-pay, and free care (vs. private insurance) had significantly higher odds of ED-DAMA (Table 2). Regarding median household income, patients in the 0–25th percentile had higher odds of ED-DAMA compared to those in the 76th to 100th percentile, with an OR of 1.14 (95% CI: 1.11–1.16).

### Inpatient admission

Pandemic (2020) was significantly associated with higher odds of IA (OR 1.04, 95% CI 1.02–1.06). Black and Native American individuals had lower odds of IA during ED visits compared to White individuals, while Hispanic and Asian individuals had higher odds of IA during ED visits compared to White individuals (Table 2). Medicaid and free care (vs. private insurance) were significantly associated with higher odds of IA. In contrast to ED-DAMA, self-pay admission was significantly associated with lower odds of IA (Table 2). The interaction between the year 2020 (pandemic) and health insurance revealed that Medicaid and self-pay admissions showed a significant increase in the odds of IA. Conversely, this interaction was negatively associated with free care (Table 2). Age was a significant factor, with varying odds across different age groups. Patients aged 12–17 (OR = 0.40, 95% CI: 0.37–0.42), 18–24 (OR = 0.54, 95% CI: 0.52–0.55), and 25–34 (OR = 0.71, 95% CI: 0.70–0.72) had lower odds of IA compared to the reference group (35–44 years). Regarding median household income, patients in the 26th to 50th percentile had lower odds of IA compared to those in the 76th to 100th percentile, with an OR of 0.97 (95% CI: 0.96–0.98).

## Discussion

Sociodemographic factors have been associated with emergency department visits (13–21). The current study investigated socioeconomic characteristics associated with ED-DAMA and ED-IA with substance use. In addition, their associations with the COVID-19 pandemic were also investigated. This study verifies some of the findings of prior studies on ED utilization (7) but it also has some new findings because it investigates the pandemic era as well as both the ED-DAMA and ED-IA. This simultaneous investigation found that whereas a positive relationship between socioeconomic characteristics and ED-DAMA indicates a disadvantaged population, this may not always be the case for ED-IA.

In the current study, gender and hospital types were similarly associated with ED-DAMA and ED-IA with substance use. Men vs. women had higher odds of both types of ED utilization, reflecting higher numbers of men with substance use visiting ED (13, 14). There was similarity in hospital type distribution. Rural hospitals had lower odds of both ED-DAMA and ED-IA. Similarly, Ibrahim et al. studied ED-DAMA using 2002 data from Nationwide Inpatient Sample and found its lower odds in rural hospitals (13). Consistent with prior publications (15, 16), rural hospitals also had lower odds of ED-IA, possibly due to the lower capacity of rural hospitals. More studies are required to explore the underlying reasons for the association between hospital characteristics and ED visit outcomes, particularly for ED-DAMA.

Here, race/ethnicity was differently associated with ED-DAMA and ED-IA with substance use. Asian patients (vs. White patients) exhibited lower odds of ED-DAMA, which might be related to higher levels of education among Asian patients (17). Another study indicated that race/ethnicity was a predictor of ED-DAMA, with higher odds for Black race (13). Tsai et al. analyzed 2019 Nationwide Emergency Department Sample data, encompassing 33,147,251 visits to 989 hospitals, to investigate disparities in ED-DAMA (7). They found that ED-DAMA odds were higher for Black and Hispanic patients compared with White patients. After adjusting for sociodemographic characteristics and hospital factors, ED-DAMA disparities reversed, with Black and Hispanic patients having lower odds of ED-DAMA compared with White patients (7). Our model was also adjusted for both demographic and hospital factors. In contrast to ED-DAMA, ED-IA showed higher odds for Asian patients as well as Hispanic patients. Black and Native American patients had lower IA odds compared to white patients in the current study. Black patients were previously found to be less likely than white patients to be admitted to the hospital after ED visits, probably related to their lack of access to regular primary care or outpatient care; therefore, they tend to go to ED for ambulatory care-sensitive conditions (18, 19).

We also found that Medicaid and free care (vs. private insurance) were significantly associated with higher odds of both ED-DAMA and ED-IA with substance use. In contrast to ED-DAMA, self-pay admission was significantly associated with lower odds of IA, which might be related to the inability of self-pay patients to afford hospital costs. Other studies also indicated that ED-DAMA odds were higher for those with no insurance or Medicaid compared to those with private insurance (13). Insurance has also previously been found to be a significant predictor of ED-IA (21).

In the current study among ED-DAMA and ED-IA with substance use, household incomes were differently associated with these ED outcomes. The lowest household income percentiles (0–25) had higher odds of ED-DAMA, indicating potential financial barriers to adhering to medical care. Household income percentiles (26–50) had lower odds of ED-IA, which might be related to the financial strains of this group. The lowest income percentile (0–25) may have access to federal and state support, which is not typically available to middle-income households (26–50).

In our study, all age groups (except 45–54), compared to the 35–44 group, had lower odds of ED-DAMA with substance use. In a systematic analysis, the mean age of ED-DAMA before 2000 was around 35 years old; however, after 2000, studies showed a mean age of ED-DAMA around 58 years old (5), indicating the complex interaction of ED-DAMA with age. Generally, being of young age (<40 years) was found to be predictive factors for ED-DAMA (5, 13). Here, for ED-IA with substance use, age groups less than 35 and over 45 had lower and higher odds compared to the reference group (35–44 years), which might reflect the better overall health status of younger patients.

Here, among ED visits with substance use, the pandemic was associated with a non-significant increase in the odds for ED-DAMA, but it was significantly associated with higher odds for ED-IA. During the pandemic, widespread fear of virus exposure, overwhelmed healthcare facilities, and shifts in hospital policies (e.g., stricter visitation and isolation protocols) may have discouraged patients from staying for full treatment, contributing to observed ED-DAMA trend. Simultaneously, increased mental health strain, substance use, and reduced access to primary care and outpatient services could have led to a higher need for inpatient care, explaining the increased odds of ED-IA (4, 8, 11). Moreover, interaction of the pandemic with race/ethnicity was only significant for Hispanic patients, with lower ED-DAMA odds for Hispanic patients, which is a favorable outcome. Therefore, our findings did not support those racial/ethnic disparities regarding ED-DAMA and ED-IA with substance use becoming worse during the COVID-19 pandemic. At the beginning of the pandemic, racial/ethnic disparities in emergency care were especially pronounced, disproportionately affecting Black, Hispanic, and American Indian/Alaska Native populations (22). However, as the pandemic progressed, these disparities in ED utilization appeared to lessen, likely due to healthcare system adaptations and broader access efforts (22). Our study, which covers the full year of 2020, might reflect this shift over time. The interaction between the pandemic and health insurance revealed that Medicaid and self-pay were significantly associated with higher odds of ED-IA. Conversely, this interaction was negatively associated with free care. Another study in California among ED visits found that the proportion of patients with private insurance increased, while Medicaid visits decreased during the pandemic (22). Our results support that the COVID-19 pandemic affected the association of health insurance with ED-IA (but not with ED-ED-DAMA) with substance use, which can be important from a policy perspective to support self-pay patients during crises, such as a pandemic.

Despite the strengths, limitations exist for the current study. Due to the cross-sectional nature of the data, this study cannot establish causal relationships between socioeconomic factors, substance use, and ED outcomes, limiting the ability to infer directional effects. The study’s retrospective design may introduce biases due to their reliance on existing data, potentially overlooking important confounding variables or causal relationships (23). Furthermore, reliance on administrative records in the current study may lead to underreporting or misclassification of certain variables, potentially affecting the accuracy of the findings. Another limitation of this study is the lack of investigation into the underlying reasons for ED visits, which might be the reason for not being admitted to the hospital. Also, other important confounding factors, such as comorbidities and disease severity, may also influence emergency medical outcomes. To address these limitations, future research should consider incorporating these factors to minimize potential biases and provide a more comprehensive understanding of the findings.

Understanding and addressing unnecessary or preventable ED visits among socioeconomically disadvantaged patients is crucial. These visits often stem from barriers to accessing primary care, lack of health education, limited resources, and systemic inequalities (4, 19). Socioeconomic factors such as poverty, unemployment, and lack of health insurance contribute to these disparities, leading individuals to rely on the ED for routine care or conditions that could be managed in outpatient settings if adequate resources and support were available (19, 24). Furthermore, cultural and language barriers, transportation issues, and stigma surrounding seeking healthcare may also contribute to the overutilization of ED services among disadvantaged populations (25). Therefore, interventions aimed at improving access to primary care, addressing social determinants of health, providing culturally competent care, and implementing community-based initiatives are essential to reduce unnecessary ED visits and improve health outcomes for socioeconomically disadvantaged individuals and communities.

## Conclusion

This study highlights the significant associations between socioeconomic characteristics and ED outcomes—specifically (ED-DAMA) and (ED-IA)—among visits involving substance use. Factors such as race/ethnicity, insurance type, income, and hospital location were found to be associated with these outcomes, with differences in how they influence DAMA versus IA. Notably, the COVID-19 pandemic modified some of these associations, particularly with regard to insurance type. These findings underscore the need for targeted healthcare interventions to address the socioeconomic barriers that contribute to suboptimal ED outcomes. Practical solutions may include general patient education, expanding access to culturally competent substance use treatment programs, improving Medicaid reimbursement policies to reduce DAMA risk, enhancing care coordination through community-based case management, and increasing the availability of peer navigators in emergency departments. Tailoring interventions to meet the needs of vulnerable populations is essential to reducing disparities, improving continuity of care, and promoting equitable health outcomes across emergency care settings.

## Data Availability

The data analyzed in this study is subject to the following licenses/restrictions: this dataset needs permission for access. Requests to access these datasets should be directed to https://hcup-us.ahrq.gov/.
